# Dysregulation of dopamine neurotransmission in the nucleus accumbens in immobilization-induced hypersensitivity

**DOI:** 10.3389/fphar.2022.988178

**Published:** 2022-09-08

**Authors:** Yuki Kishikawa, Yukie Kawahara, Yoshinori N. Ohnishi, Naoki Sotogaku, Tomoko Koeda, Hiroshi Kawahara, Akinori Nishi

**Affiliations:** ^1^ Department of Rehabilitation Sciences, Faculty of Rehabilitation Sciences, Nishikyushu University, Kanzaki, Japan; ^2^ Department of Pharmacology, Kurume University School of Medicine, Kurume, Japan; ^3^ Department of Physical Therapy, Faculty of Rehabilitation Sciences, Nagoya Gakuin University, Nagoya, Japan; ^4^ Department of Dental Anesthesiology, Tsurumi University School of Dental Medicine, Yokohama, Japan

**Keywords:** postsynaptic, hyperalgesia, microdialysis, rat, D2-like receptor

## Abstract

Cast immobilization causes sensory hypersensitivity, which is also a symptom of neuropathic pain and chronic pain. However, the mechanisms underlying immobilization-induced hypersensitivity remain unclear. The present study investigated the role of dopamine neurotransmission in the nucleus accumbens shell (NAcSh) of rats with cast immobilization-induced mechanical hypersensitivity using *in vivo* microdialysis. Cast immobilization of the hind limb decreased the paw withdrawal threshold (PWT). Mechanical stimulation of the cast-immobilized hind limb induced a decrease in dopamine in the NAcSh, and this decrease was associated with the upregulation of presynaptic D2-like receptors. A D2-like receptor antagonist infused into the NAcSh reversed the decrease in PWT in rats with cast immobilization, whereas a D2-like receptor agonist infused into the NAcSh induced a decrease in PWT in control rats. In addition, the expression of the D2 receptor (*Drd2*) mRNA in the NAcSh was increased by cast immobilization. Importantly, systemic administration of the D2-like receptor antagonist reversed the decrease in PWT in rats with cast immobilization. As dopamine levels regulated by presynaptic D2-like receptors did not correlate with the PWT, it is presumed that the D2-like receptor antagonist or agonist acts on postsynaptic D2-like receptors. These results suggest that immobilization-induced mechanical hypersensitivity is attributable to the upregulation of postsynaptic D2-like receptors in the NAc. Blockade of D2-like receptors in the NAcSh is a potential therapeutic strategy for immobilization-induced hypersensitivity.

## 1 Introduction

Limb immobilization induces sensory hypersensitivity in humans (Terkelsen et al., 2008) and animals (Nakano et al., 2012; Hamaue et al., 2013; Sekino et al., 2014; Nakagawa et al., 2018), which is also involved in mechanisms of allodynia and hyperalgesia in neuropathic pain (Jensen and Finnerup, 2014). In neuropathic pain, nerve injury induces neuroplastic changes *via* multiple mechanisms along nociceptive pathways, which extend from the periphery to the central nervous system, including the spinal cord, brain, and descending modulatory pathways (Cohen and Mao, 2014). Similar mechanisms are assumed to contribute to the development and maintenance of immobilization-induced hypersensitivity. Immobilization of hind limb induces epidermal thinning, the increased nerve fibers, especially myelinated A fibers and unmyelinated C fibers, in the epidermis and upper dermis and the increased expression of pain-related molecules such as nerve growth factor, transient receptor potential vanilloid 1 and P2X_3_ in the epidermis (Nakano et al., 2012; Sekino et al., 2014), and the changes in skin tissues may be related to sensory hypersensitivity. Maladaptive central changes, such as spinal sensitization, due to an elevated number of wide-dynamic-range neurons (Ushida and Willis, 2001) and high expression of calcitonin gene-related peptide in the spinal dorsal horn (Nishigami et al., 2009; Hamaue et al., 2013) are also reported. However, supraspinal brain mechanisms underlying immobilization-induced hypersensitivity are not well understood.

Chronic pain affects brain networks, including the mesolimbic dopamine system, which plays a critical role in the perception and modulation of chronic pain (Baliki et al., 2012; Mitsi and Zachariou, 2016; Serafini et al., 2020). In rodent models of neuropathic pain, it has been shown that the activity of dopaminergic neurons in the ventral tegmental area (VTA) and the release of dopamine in the nucleus accumbens shell (NAcSh) are suppressed. Additionally, adaptation of D2-type/indirect pathway medium spiny neurons (MSNs) to the hypodopaminergic state is involved in mechanisms of central hypersensitivity in chronic pain (Ren et al., 2016). Several reports have demonstrated that neuropathic pain is amplified by the increased excitability of D2-type MSNs in the NAcSh due to low dopamine/D2 receptor inactivation or chemogenetic activation, and is alleviated by the decreased excitability of D2-type MSNs due to D2-like receptor activation, infusion of lidocaine, or chemogenetic inhibition (Sarkis et al., 2011; Chang et al., 2014; Ren et al., 2016; Harris and Peng, 2020). However, injection of a local anesthetic, bupivacaine, into the NAc core enhances the nociceptive effect of formalin (Magnusson and Martin, 2002). These controversial results hint at the complexity of processing pain by the neural circuit involving the NAc shell and core. Furthermore, the modulatory role of dopamine neurotransmission switches from antinociceptive to pronociceptive during the transition from acute to chronic pain (Dias et al., 2015). Plastic changes in the dopamine system need to be evaluated in each neural circuit for pain control and in the developmental stage of chronic pain.

Despite the importance of the mesolimbic dopamine system in chronic pain, there is no direct evidence of altered dopamine neurotransmission in the NAc under immobilization-induced hypersensitivity. Within the NAc, the NAcSh is a key node that receives the projection from VTA dopamine neurons and plays important roles in pain control (Ren et al., 2016) as well as reward (Di Chiara and Bassareo, 2007) and aversion (Badrinarayan et al., 2012). Therefore, we investigated the role of dopamine neurotransmission in the NAcSh of rats with chronic cast immobilization. Pharmacological analyses revealed that immobilization-induced hypersensitivity is mediated through upregulation of D2-like receptor signaling, and that blockade of D2-like receptors expressed on MSNs in the NAcSh attenuates hypersensitivity.

## 2 Materials and methods

### 2.1 Animals

Male Wistar rats (SLC, Shizuoka, Japan) aged 7–12 weeks (210–310 g) were used for experiments. Two rats per cage (40 × 25 × 20 cm) were maintained on a 12/12 h light/dark cycle at a temperature of 21 ± 2°C with wood shaving floor and free access to standard food/water in conventional area. Welfare-related assessments and interventions were carried out prior to and during the experiment. Forty-eight rats with cast immobilization and 53 control rats were used to measure extracellular dopamine levels by microdialysis (Supplementary Figure S1); 27 rats with cast immobilization and 13 control rats were used to measure paw withdrawal threshold (PWT); and 7 rats with cast immobilization and 19 control rats were used to measure both extracellular dopamine and PWT. Plaster cast application procedure, von Frey test for mechanical hypersensitivity, surgery for brain microdialysis and dissection of brain for quantitative real-time PCR were carried out during 12 h-light period in conventional area. Sixteen rats with cast immobilization and 15 control rats were used for quantitative real-time PCR. No adverse events were observed excluding the events for experimental purpose [reduction of PWT and range of motion (ROM)]. Considering that the development of chronic pain is related to differences of sexual hormones between the sexes, only male rats were used in this study (Sorge and Totsch, 2017). All rats were handled in accordance with the Guide for the Care and Use of Laboratory Animals, as adopted and promulgated by the United States. National Institutes of Health, and the protocols were approved by the Institutional Animal Care and Use Committee of Kurume University School of Medicine. All efforts were made to minimize animal suffering and limit the number of animals used.

### 2.2 Drugs

A dopamine D2-like receptor antagonist, S (−)-raclopride (Sigma-Aldrich, St. Louis, MO), and a D2-like receptor agonist, (−)-quinpirole HCL (Research Biochemicals International, Natick, MA), were dissolved in Ringer’s solution (NaCl 140 mM, KCl 3 mM, MgCl2·6H_2_O 1 mM, CaCl2·2H_2_O 1.2 mM) for application by retrograde microdialysis. Raclopride was dissolved in saline for intraperitoneal administration.

### 2.3 Plaster cast application procedure

Rats were habituated to the animal facility for 1 week from their arrival. Eight-week-old rats were anesthetized with sodium pentobarbital (50 mg/kg), and subjected to unilateral cast immobilization. The left hind limbs of the rats were encased in plaster casts (ALCARE, Tokyo, Japan) in a full plantar flexed position. After casting, the rats were housed one per cage for 4 weeks. The left hind limb was immobilized for 4 weeks to measure dopamine in the chronic state 2 weeks after reaching maximum effects on the PWT (Nakano et al., 2012; Sekino et al., 2014). In addition, 4 weeks of immobilization was chosen based on the fact that the function of dopamine neurotransmission was significantly changed in the late phase (approximately 4 weeks) of neuropathic pain (Chang et al., 2014; Kato et al., 2016). The cast was changed every week, during which time the body weight, general conditions of the hind limb visually inspected and the ROM of the ankle joint were evaluated (Honda et al., 2021). Briefly, the ROM was measured as the angle (0°–180°) between the line connecting the fifth metatarsal to the malleolus lateralis of the fibula and the line connecting the malleolus lateralis of the fibula to the center of the knee joint. The ROM in the control rats was 170°, which was set at 100%. The control rats were housed one per cage for 4 weeks, and the ROM of the ankle joint was measured every week.

### 2.4 Von Frey test for mechanical allodynia

Von Frey hair was used to test for secondary allodynia (Philpott et al., 2017). Alert, unanesthetized animals were placed in a chamber with a metal mesh flooring, which allowed access to the plantar surface of each hind paw. After allowing the animals to acclimate until exploratory behavior ceased (approximately 10 min), ipsilateral (left) hind paw mechanosensitivity was assessed using a modification of the Dixon up-down method (Dixon, 1965). A series of eight calibrated von Frey filaments, ranging from 0.4 to 15 g in log increments, were used. The test was initiated with a 2.0 g von Frey filament, the middle of the series of filaments, applied to the plantar surface of the ipsilateral hind paw for 7–10 s. Von Frey hair was applied perpendicular to the plantar surface of the ipsilateral hind paw until the hair flexed. Beside the Dixon up-down method, a von Frey filament with 30 g bending force was applied to control rats, since the von Frey filament with 30 g bending force was required to initiate the response in control rats. Tactile sensitivity is usually expressed as the PWT. PWT was measured as the hind paw withdrawal response to von Frey filament stimulation. If there was a positive response, the next lower strength hair was applied, and if there was no response, the next higher strength hair was applied up to a cut-off of 15 g bending force. The 50% withdrawal threshold was determined using the following formula: 10 (Xf + kδ)/10,000; where Xf = value (in log units) of final von Frey hair used, k = tabular value for the pattern of the last six positive and/or negative responses, and *δ* = mean difference (in log units) between stimuli (Chaplan et al., 1994). The von Frey test was performed once weekly for 4 weeks after cast immobilization, 2 h after drug infusion or 1 h after intraperitoneal administration. In pilot studies, the effects of cast immobilization on the PWT were not observed on the contralateral side as reported in previous studies (Nakano et al., 2012; Hamaue et al., 2013; Chuganji et al., 2015).

### 2.5 Surgery and brain microdialysis

Following a period of 4 weeks of cast immobilization of the ipsilateral hind limb, approximately 40 min surgery was conducted in the fifth week in 12-week-old rats under pentobarbital (50 mg/kg i.p.) and xylazine (8 mg/kg i.p.) anesthesia and local application of 10% lidocaine. Probes were implanted in the right (contralateral) side of the NAcSh (exposed length, 1 mm) or prefrontal cortex (PFC; exposed length, 6 mm). Dopamine was measured on the right side of the brain, because one side of hind limb immobilization mainly affects the contralateral side of the brain (Viaro et al., 2014). The coordinates of probe implantation were A/P, 1.7 mm; L/M, 0.8 mm; and V/D, 7.2 mm, from the bregma and dura for the NAcSh region; A/P, 2.5 mm and L/M, 2.0 mm, from the bregma; and V/D, 6.0 mm from the dura at an angle of 14° in the coronal plane of the PFC (Paxinos, 2001; Kawahara et al., 2013; Kaneko et al., 2016). After surgery, the rats were housed individually for recovery 24–48 h before microdialysis experiments. The rats were moved to an acrylic box (30 × 30 × 40 cm), and microdialysis probes were placed in the guide cannulas. Dialysate fractions were collected every 20 min. The flow rate of Ringer’s solution was 2.0 μl/min. Dopamine was quantified by High Performance Liquid Chromatography (HPLC) with electrochemical detection conducted as described in our previous study (Kawahara et al., 2009). At the end of the experiments, the rats were given an overdose of chloral hydrate, and their brains were fixed with 4% paraformaldehyde *via* intracardiac infusion. Coronal sections (50 μm thickness) were cut, and dialysis probe placement was localized based on the atlas of Paxinos et al. (2007). Rats in which dialysis probes and guide cannulas were misplaced were not included in the data analyses (*n* = 3).

### 2.6 Stimulation method with von Frey filament

Von Frey filament stimulation of the ipsilateral hind limb was applied once every 30 s for 20 min during the measurement of dopamine by *in vivo* microdialysis. The intensity of von Frey filament stimulation was 30 g for the control group and 6 g or 0.4 g for the cast group. The 30 and 6 g of von Frey filament stimulation were always strong enough to induce a paw withdrawal response in the control and cast groups, respectively.

### 2.7 Quantitative real-time PCR

Total RNA was isolated from dissected brain tissues using Sepasol RNA I Super G (Nacalai Tesque, Kyoto, Japan). After genomic DNA cleaning, cDNA was obtained by total RNA reverse transcription using a QuantiTect Reverse Transcription Kit (Qiagen, Valencia, CA). Real-time PCR was performed using a LightCycler 480 System II (Roche, Basel, Switzerland) with Gene Ace SYBR^®^ qPCR Mix α (NIPPON GENE, Toyama, Japan). The following primers were used for gene amplification: D2 receptor, sense 5′-AAG​CGC​CGA​GTT​ACT​GTC​AT-3′, anti-sense 5′-GGC​AAT​GAT​ACA​CTC​ATT​CTG​GT-3′; β-actin, sense 5′-CGT​GAA​AAG​ATG​ACC​AGA​T-3′, anti-sense 5′-ATT​GCC​GAT​AGT​GAT​GAC​CT-3′. The relative mRNA levels of the individual samples were calculated using the comparative Ct method (2^−∆∆Ct^). cDNA was detected using the LightCycler 480 SYBR Green I Master. The PCR cycles were performed at 95°C for 10 min, followed by 45 cycles of denaturation at 95°C for 30 s and annealing/extension at 60°C for 1 min.

### 2.8 Expression of data and statistical analysis

Data of the PWT and ROM are displayed as mean ± standard deviation (SD), and the rest of data are displayed as mean ± standard error of mean (SEM). For analysis of microdialysis data, all values were expressed as a percentage of the basal values (100%) for each group, obtained as the average of three stable baseline samples for dopamine. The values obtained after drug infusion or von Frey filament stimulation were compared with the basal values by mixed linear models, with time as a covariate using the SAS MIXED procedure (version 9.4, SAS Institute, Cary, NC). Data pertaining to microdialysis, PWT, ROM of the ankle joint, and quantitative real-time PCR were compared between the control and cast groups using two-way ANOVA, followed by Sidak’s multiple comparisons test. The basal values of dopamine were compared using the Welch’s *t*-test. PWT data were analyzed using Welch’s *t*-test, Mann-Whitney test or Kruskal-Wallis test, followed by Dunn’s multiple comparisons test. Analyses were performed using Prism 8 software (GraphPad Software, San Diego, CA). Statistical significance was set at *p* < 0.05. Details of the statistical analyses are listed in Supplementary Tables S2–S6.

## 3 Results

### 3.1 Effects of cast immobilization of hind limb on the paw withdrawal threshold and ROM of ankle joint dorsiflexion

**FIGURE 1:**
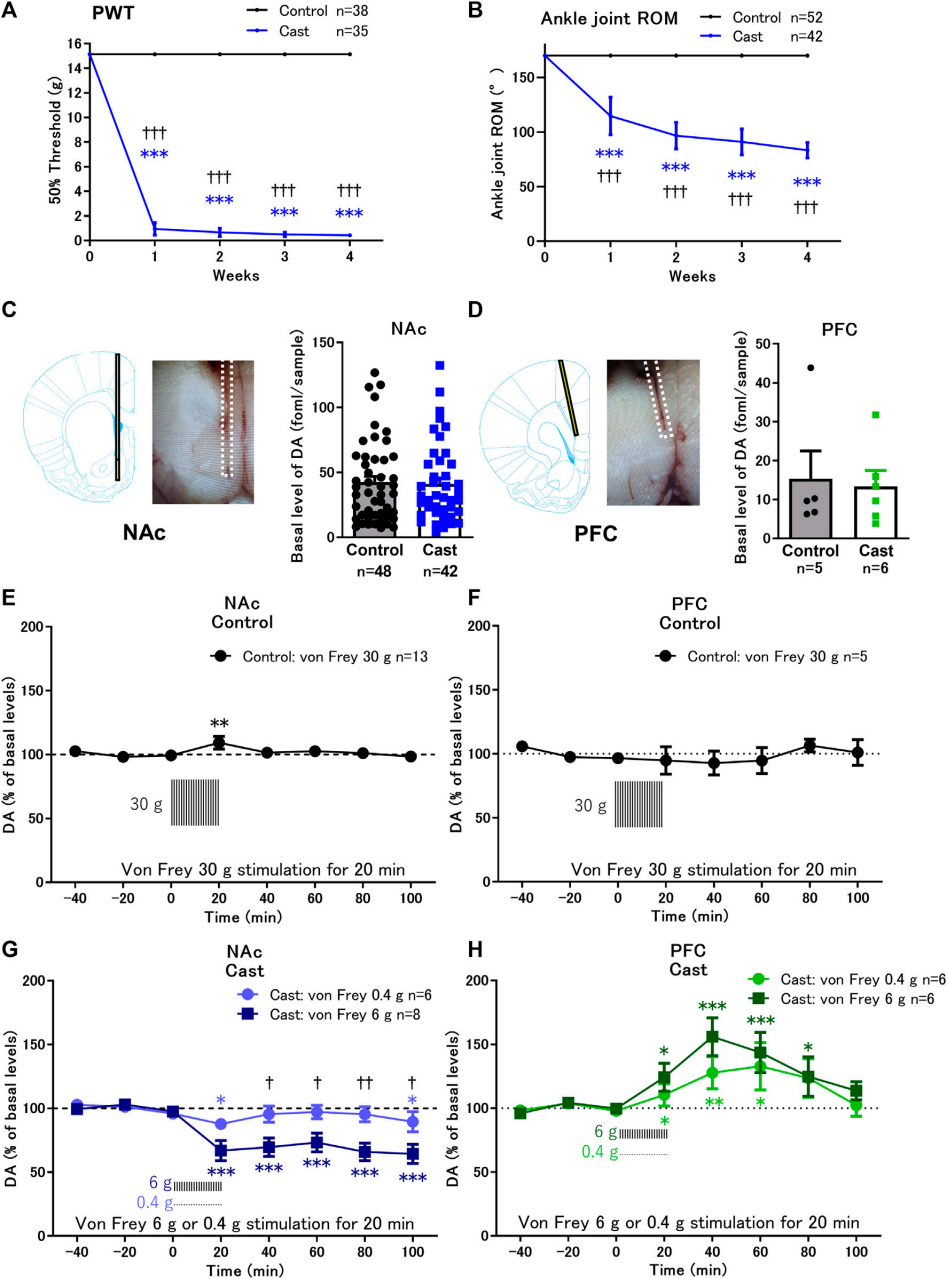
Effects of cast immobilization of the left hind limb on the paw withdrawal threshold (PWT), range of motion (ROM), and von Frey filament stimulation-induced changes of dopamine (DA) in the nucleus accumbens shell (NAcSh) and prefrontal cortex (PFC). **(A,B)** Cast immobilization significantly decreased the PWT **(A)** and ROM of the ankle joint **(B)** in the cast group. ***p < 0.001 versus PWT and ROM at 0 week; ^†††^ *p* < 0.001 versus the control group; two-way ANOVA and Sidak’s multiple comparisons test. **(C,D)** Representative traces of microdialysis probe placed in the NAcSh **(C)** or PFC **(D)** are indicated. Basal levels of extracellular DA in the NAcSh **(C)** and PFC **(D)** in the cast group were similar to those in the control group. **(E,G)** In the NAcSh, von Frey filament stimulation induced a slight increase in DA in the control group **(E)** but induced a significant decrease in DA in the cast group **(G)**. **(F–H)** In the PFC, von Frey filament stimulation did not affect the DA levels in the control group **(F)** but increased the DA levels in the cast group **(H)**. The basal levels for each group were obtained as the average of three stable baseline samples (100%), and all values were calculated as a percentage of the basal values within the same group. *p < 0.05, **p < 0.01, ***p < 0.001 versus the basal DA levels; mixed linear models. ^†^ *p* < 0.05, ^††^ *p* < 0.01 between von Frey stimulation 6 and 0.4 g in the cast group; two-way ANOVA and Sidak’s multiple comparisons test. Data represent mean ± SD **(A,B)** and mean ± SEM **(C–H)**. The number of rats is indicated in the graph.

Rats with cast immobilization of the left hind limb (the cast group) showed a marked decrease in PWT, compared with the control group (Figure 1A). The PWT remarkably decreased 1 week after cast placement. The ROM of ankle joint dorsiflexion significantly decreased 1 week after cast placement, compared with the control group, and continuously decreased during the 4 weeks of cast immobilization (Figure 1B). The results of ROM with cast immobilization were considered as an indication for proper condition of cast immobilization.

### 3.2 Alteration of dopamine response to von Frey stimulation in the NAcSh and PFC of rats with cast immobilization

Basal levels of extracellular dopamine in the contralateral NAcSh (Figure 1C) and PFC (Figure 1D) in rats with cast immobilization for 4 weeks were similar to those in the control group (Supplementary Table S1). Von Frey filament stimulation of the cast-immobilized hind limb was applied to the control and cast groups once every 30 s for 20 min. In the control group, rats were stimulated with 30 g von Frey filament to induce paw withdrawal response in all rats. Under these conditions, von Frey stimulation induced a small increase in dopamine levels in the NAcSh (Figure 1E), but not in the PFC (Figure 1F). In the cast group, rats were stimulated with 0.4 g or 6 g von Frey filament. Von Frey stimulation (6 g) continuously decreased dopamine levels to approximately 60% of the basal levels in the NAcSh from 20 to 100 min, although the effects of 0.4 g von Frey stimulation were marginal (Figure 1G). In contrast, in the PFC, 0.4 and 6 g von Frey stimulation induced brief increases in dopamine levels, with maximal increase at 40–60 min (Figure 1H).

The increase in dopamine levels in response to various stressors in the PFC has been extensively studied (Thierry et al., 1976). However, the role of dopamine neurotransmission in the NAcSh in immobilization-induced hypersensitivity is not fully understood. Therefore, we focused on the NAcSh to further analyze the mechanism of the decreased dopamine levels in response to von Frey stimulation and the role of dopamine in the decreased PWT in a rat model of cast immobilization.

### 3.3 Effects of local infusion of raclopride on the decrease of dopamine levels in the NAcSh in response to von Frey stimulation

**FIGURE 2:**
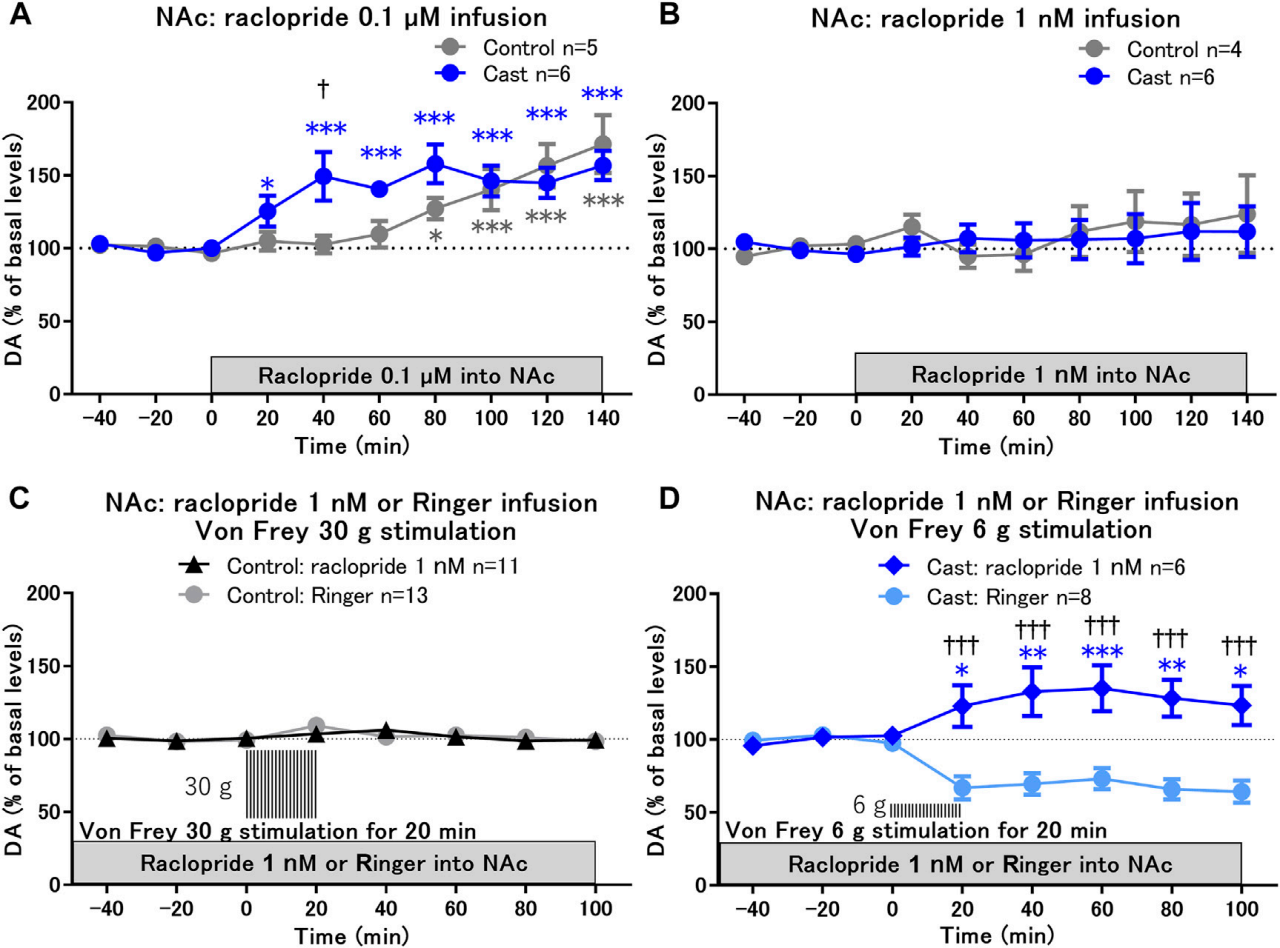
Effects of local infusion of raclopride on von Frey stimulation-induced changes in dopamine (DA) levels in the nucleus accumbens shell (NAcSh) of rats with cast immobilization. **(A,B)** Effects of local infusion of a D2-like receptor antagonist, raclopride, into the NAcSh at 0.1 μM **(A)** and 1 nM **(B)** on the basal levels of DA; *p < 0.05, ***p < 0.001 versus the basal levels of DA in the same group; mixed linear models; ^†^ *p* < 0.05 between the control and cast groups; two-way ANOVA and Sidak’s multiple comparisons test. **(C,D)** Von Frey filament stimulation-induced changes of DA in the NAcSh after 2 h of local infusion of raclopride at a low dose (1 nM) in the control **(C)** and cast **(D)** groups. DA levels for the first 80 min of raclopride infusion are not included, because raclopride infusion at 1 nM did not affect DA levels for 120 min as shown in **(B)**. Results of von Frey filament stimulation with Ringer infusion in the control **(C)** and cast **(D)** groups were reproduced from Figures 1E,F, respectively. *p < 0.05, **p < 0.01, ***p < 0.001 versus the basal levels of DA in the same group; mixed linear models; ^†††^ *p* < 0.001 between groups with Ringer and raclopride infusion; two-way ANOVA and Sidak’s multiple comparisons test; Data represent mean ± SEM. The number of rats is indicated in the graph.

We first evaluated whether D2-like receptor function in the NAcSh was altered in rats with cast immobilization. Infusion of a D2-like receptor antagonist, raclopride, at 0.1 μM into the NAc, but not at 1 nM, induced increases in dopamine levels in the NAcSh in the control and cast groups (Figures 2A,B). The increases in dopamine levels, especially in the early phase of raclopride infusion at 40 min, were significantly higher in the cast group than that in the control group (Figure 2A). The results suggest that the function of D2-like receptors to inhibit dopamine release from dopaminergic terminals (Benoit-Marand et al., 2001) is enhanced in rats with cast immobilization, although basal dopamine levels were not altered in accordance with previous studies (Kennedy et al., 1992; Beckstead et al., 2007; Benoit-Marand et al., 2011).

To study the role of D2-like receptors in the decreased dopamine levels in response to von Frey stimulation in the cast group, the effects of von Frey stimulation on dopamine levels were examined under conditions of raclopride infusion at a low dose of 1 nM, at which raclopride alone did not affect the basal levels of dopamine (Figure 2B). After 2 h of raclopride infusion with the stable baseline of dopamine, hind paw stimulation with 30 g von Frey filament for 20 min did not affect dopamine levels in the control group (Figure 2C). In contrast, in the cast group, raclopride infusion induced a shift in dopamine levels from a decrease to an increase in response to 6 g von Frey stimulation (Figure 2D). These results suggest that the decrease in dopamine in response to mechanical stimulation is mediated through the upregulation of presynaptic D2-like receptor function in the NAcSh of rats with cast immobilization.

### 3.4 Effects of local infusion of raclopride and quinpirole into the NAcSh on the paw withdrawal threshold

**FIGURE 3:**
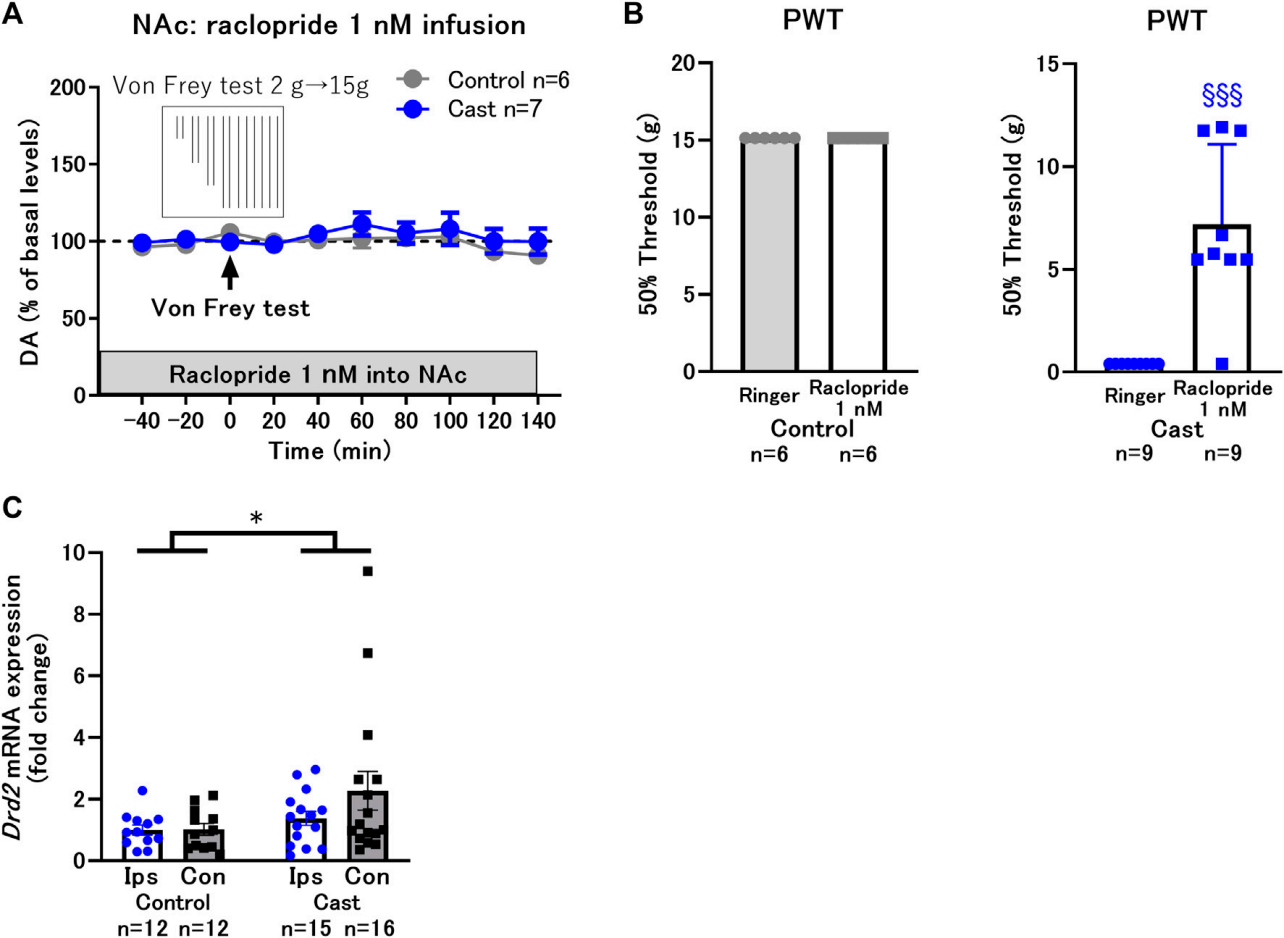
Effects of local infusion of raclopride into the nucleus accumbens shell (NAcSh) on the decreased paw withdrawal threshold (PWT) in rats with cast immobilization. **(A)** Effects of the von Frey test after 2 h of local infusion of raclopride at a low dose (1 nM) on DA levels in the control and cast groups. **(B)** Local infusion of raclopride at a low dose (1 nM) did not affect the PWT in the control group, but drastically increased it in the cast group. ^§§§^ *p* < 0.001 versus the group with Ringer infusion; Welch’s *t*-test. **(C)** Effects of cast immobilization on the expression of *Drd2* mRNA in the ipsilateral (Ips) and contralateral (Con) NAcSh. These results were obtained from different experimental groups from **(A)** and **(B)**, and did not go through the microdialysis experiments or drug infusions. Therefore, there were no tissue damages from the microdialysis probe implantations. Homogeneity of variance was evaluated with Levene’s test (*p* = 0.1008) and the result implicate that variances are equal across groups. *p < 0.05 between the control and cast group; two-way ANOVA. Data represent mean ± SEM **(A,C)** and mean ± SD **(B)**. The number of rats is indicated in the graph.

Next, we examined whether the upregulation of D2-like receptor function in the NAcSh contributes to the hypersensitivity of paw withdrawal response in rats with cast immobilization. After blockade of D2-like receptors in the NAcSh with raclopride infusion at 1 nM for 2 h, the von Frey test was performed. Under these conditions, the von Frey test did not affect dopamine levels in either control or cast group (Figure 3A). Raclopride infusion did not affect the PWT in the control group, although the changes in the PWT in a range larger than 15 g bending force were not determined. In contrast, raclopride infusion increased it in the cast group (Figure 3B). The results suggest that the upregulation of D2-like receptor function is critical for the decrease in PWT in a rat model of cast immobilization. As the decreased PWT in the cast group was mediated by upregulation of D2-like receptor function, the expression of D2 receptor (*Drd2*) mRNA was analyzed in NAcSh tissues after 5 weeks of cast immobilization. In the cast group, cast immobilization induced an increase in *Drd2* mRNA compared with the control group, although a significant difference in *Drd2* mRNA between the contralateral and ipsilateral sides of the NAcSh was not detected in the cast group (Figure 3C).

**FIGURE 4:**
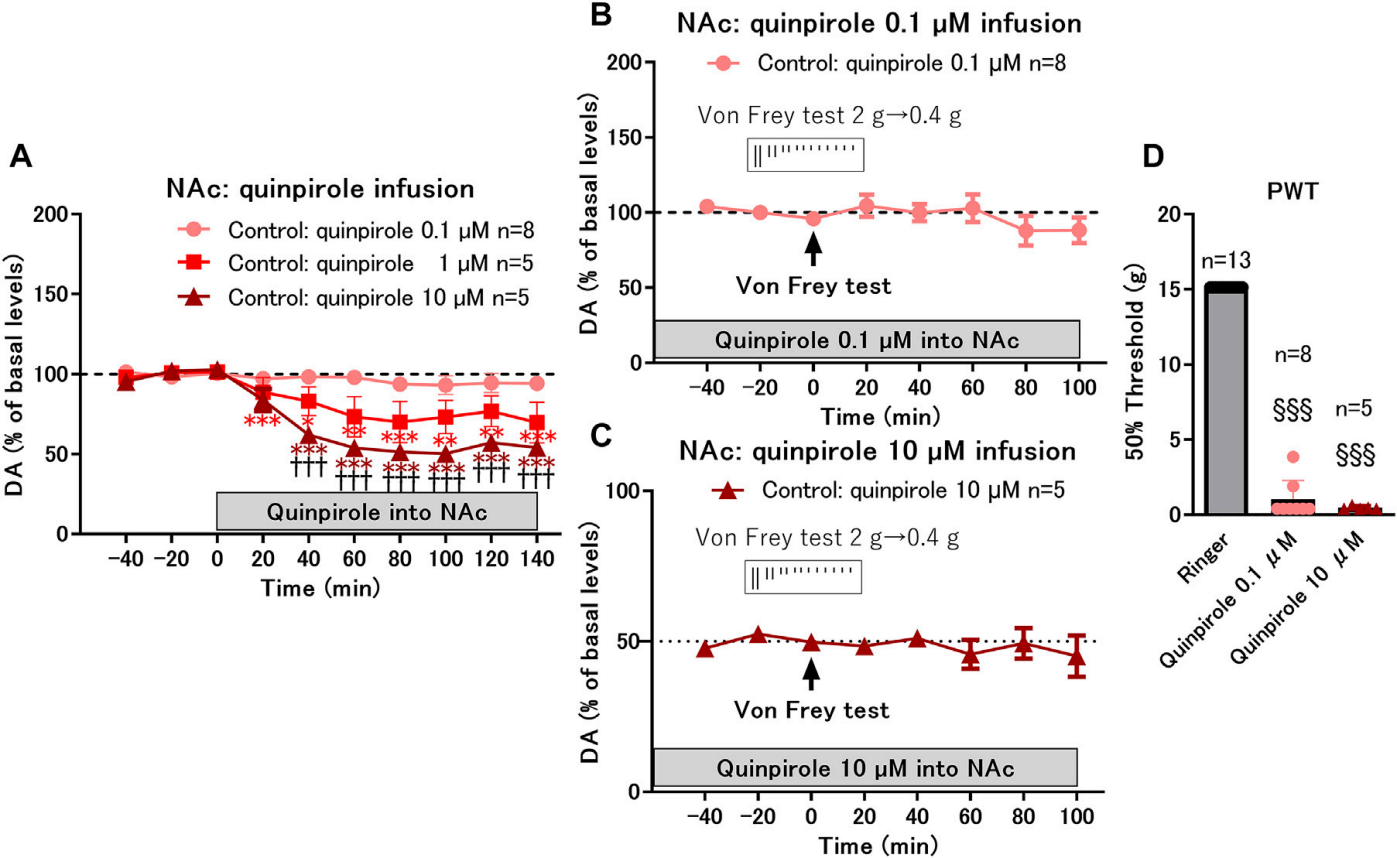
Effects of local infusion of quinpirole into the nucleus accumbens shell (NAcSh) on the paw withdrawal threshold (PWT) in control rats. **(A)** Effects of local infusion of a D2-like receptor agonist, quinpirole, into the NAcSh (0.1, 1, and 10 μM) on the basal levels of DA in the control group; *p < 0.05, **p < 0.01, ***p < 0.001 versus the basal levels of DA in the same group; mixed linear models; ^†††^ *p* < 0.001 between 10 and 0.1 μM groups; two-way ANOVA and Sidak’s multiple comparisons test. **(B,C)** Effects of von Frey test after 2 h of local infusion of quinpirole at a low dose (0.1 μM) with constant dopamine levels (−100% of basal levels) **(B)** and at a high dose (10 μM) with decreased dopamine levels (−50% of basal levels) **(C)** on DA levels; **(D)** Local infusion of quinpirole at low (0.1 μM) and high (10 μM) doses drastically decreased the PWT. ^§§§^ *p* < 0.001 versus the group with Ringer infusion; Kruskal-Wallis test and Dunnet’s multiple comparisons test. Data represent mean ± SEM **(A–C)** and mean ± SD **(D)**. The number of rats is indicated in the graph.

To mimic the upregulated state of D2-like receptor function in rats with cast immobilization, a D2-like receptor agonist, quinpirole, was infused into the NAcSh in the control group. Infusion of quinpirole at 0.1 μM did not affect dopamine levels; however, infusion at 1 and 10 μM decreased dopamine levels in a dose-dependent manner (Figure 4A). The von Frey test was performed after 2 h of quinpirole infusion at a low dose (0.1 μM) with constant dopamine levels (−100% of basal levels) (Figure 4B) and at a high dose (10 μM) with decreased dopamine levels (−50% of basal levels) (Figure 4C). The von Frey test did not affect dopamine levels but induced a drastic decrease in the PWT after quinpirole infusion both at low and high doses (Figure 4D). These results suggest that activation of D2-like receptors in the NAcSh increases the sensitivity of paw withdrawal response to mechanical stimulation, despite constant or decreased dopamine levels. Taken together, D2-like receptor function is likely upregulated at both postsynaptic and presynaptic sites in rats with cast immobilization. However, the upregulation of D2-like receptor function at postsynaptic sites seems to be critical for the increased sensitivity, because dopamine levels regulated by presynaptic D2-like receptors do not correlate with the PWT. It is possible that upregulation of presynaptic D2-like receptors works to compensate for mechanical hypersensitivity by decreasing dopamine levels in the NAcSh after mechanical stimulation (Figure 2D).

### 3.5 Effects of systemic administration of raclopride on the decreased paw withdrawal threshold in rats with cast immobilization

**FIGURE 5:**
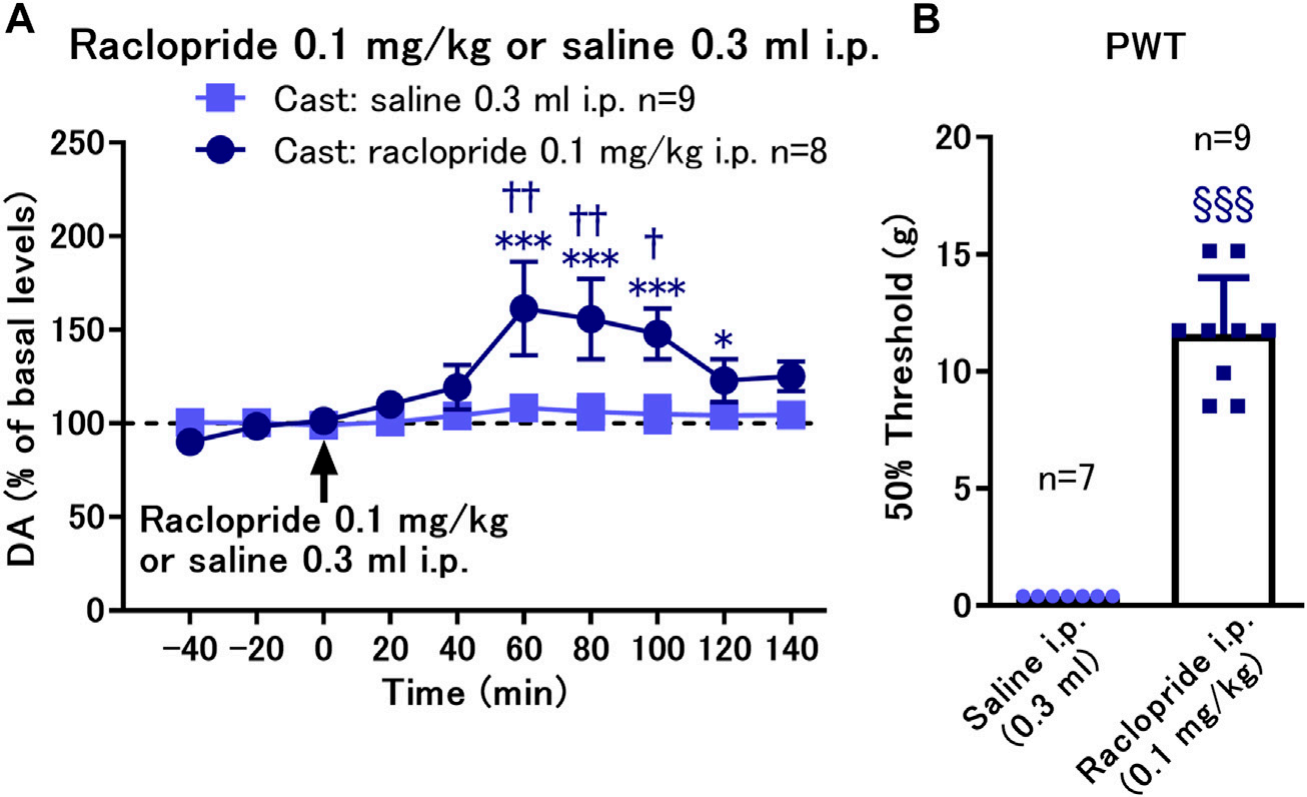
Effects of systemic administration of raclopride on the paw withdrawal threshold (PWT) in rats with cast immobilization. **(A)** Administration of raclopride 0.1 mg/kg, i.p. induced a significant increase in dopamine (DA) levels. *p < 0.05, ***p < 0.001 versus the basal levels of DA in the same group; mixed linear models; ^†^ *p* < 0.05, ^††^ *p* < 0.01 between groups with saline and raclopride administration; two-way ANOVA and Sidak’s multiple comparisons test; **(B)** Administration of raclopride reversed the decreased PWT evaluated with von Frey test 60 min after raclopride or saline administration. ^§§§^ *p* < 0.001 versus the group with saline infusion; Welch’s *t*-test; Data represent mean ± SEM **(A)** and mean ± SD **(B)**. The number of rats is indicated in the graph.

We found that D2-like receptor antagonism by local infusion of raclopride into the NAcSh reversed the decreased PWT in the cast group. Therefore, we investigated whether systemic administration of raclopride is effective in reversing the decreased PWT in the cast group for exploration of translational possibilities of the D2-like receptor antagonist. Systemic administration of raclopride (0.1 mg/kg, i.p.) induced a brief increase in dopamine levels after 60–100 min of administration (Figure 5A). The von Frey test was performed 60 min after raclopride administration when dopamine was maximally increased. Under these conditions, raclopride increased the PWT from an extremely low level in the cast group (Figure 5B), suggesting that systemic administration of the D2-like receptor antagonist is effective in improving mechanical hypersensitivity caused by cast immobilization.

## 4 Discussion

The present study demonstrated that mechanical hypersensitivity induced by chronic cast immobilization was attributable to altered dopaminergic neurotransmission in the NAcSh. *In vivo* microdialysis analyses combined with drug infusion into the NAcSh revealed that D2-like receptor blockade in the NAcSh reversed mechanical hypersensitivity, whereas D2-like receptor activation in the NAcSh mimicked mechanical hypersensitivity despite normal or low dopamine levels, suggesting that mechanical hypersensitivity is presumably mediated through upregulation of postsynaptic D2-like receptors in the NAcSh. This interpretation is supported by the increased expression of *Drd2* mRNA in the NAcSh. In addition, mechanical stimulation of the cast-immobilized hind limb induced a decrease in dopamine in the NAcSh, and upregulation of presynaptic D2-like receptors at dopaminergic terminals contributed to the decrease in dopamine. The therapeutic effectiveness of systemic administration of the D2-like receptor antagonist in a rat model of mechanical hypersensitivity encourages the development of potential therapeutics for certain types of chronic pain with D2-like receptor upregulation.

### 4.1 Role of postsynaptic D2-like receptors in immobilization-induced mechanical hypersensitivity

Pharmacological manipulation of dopamine D2-like receptor activity in the NAcSh identified that mechanical hypersensitivity induced by cast immobilization is mediated through the upregulation of postsynaptic D2-like receptors. The increased expression of *Drd2* mRNA in the NAcSh is mainly attributable to the increased expression in D2-type MSNs, rather than at dopaminergic terminals, because mRNA is more abundant in the cell body than in axons and nerve endings. Therefore, the upregulation of postsynaptic D2-like receptors is likely accompanied by an increase in D2 receptor expression in D2-type MSNs. This finding is consistent with a recent study showing increased expression of *Drd2* mRNA and D2 receptor protein in the NAc in mice with chronic constriction injury of the sciatic nerve (Wawrzczak-Bargieła et al., 2020). Interestingly, in patients with chronic orofacial pain, positron emission tomography revealed high D2-like receptor availability for the binding of [^11^C] raclopride, which indicates a high density of D2-like receptors and/or low dopamine levels in the striatum (Hagelberg et al., 2004). Thus, the upregulation of postsynaptic D2-like receptors is a mechanism for mechanical hypersensitivity as well as chronic neuropathic pain.

The mechanisms underlying the upregulation of D2-like receptor function are currently unknown. Long-term stress from the disability of the unilateral hind paw causes numerous interrelated plastic changes in muscles, connective tissues, and the central nervous system (Hamaue et al., 2013; Sekino et al., 2014; Ledri et al., 2020). In addition, mechanical stimulation has been shown to activate the functional connectivity of the NAc including shell region with cortical areas such as the PFC and anterior cingulate cortex, and these functional connectivities are enhanced with chronic pain (Baliki et al., 2010; Baliki et al., 2012; Gao et al., 2020). The cortical excitatory pathways, dominantly projected on D2-type MSNs in the NAc (Deroche et al., 2020; Gao et al., 2020), may affect D2-like receptor function. Furthermore, chronic stress, such as chronic unpredictable stress and chronic pain, has been shown to decrease dopamine levels in the NAc (Taylor et al., 2016; Qiao et al., 2020). Under conditions of a hypodopaminergic state, phasic dopamine release evoked by noxious stimuli can be augmented (Gee et al., 2020). Augmented phasic dopamine neurotransmission might act as one of the mechanisms underlying the upregulation of D2-like receptor function, as activation of D2-like receptors is shown to upregulate the expression of D2-like receptors in the rat striatum (Tokunaga et al., 2012).

Contrary to our observation, activation of dopamine neurotransmission *via* D2-like receptors has been shown to relieve pain (Taylor et al., 2016; Liu et al., 2019; Wang et al., 2021), presumably by inhibiting the excitability of D2-type MSNs in the NAcSh (Ren et al., 2016). For instance, during the acute phase of pain, dopamine neurotransmission *via* D2-like receptors in the NAc attenuates acute inflammatory pain in rats (Taylor et al., 2003; Dias et al., 2015). However, the increased activity of dopamine neurotransmission *via* D2-like and D1-like receptors facilitates the transition from acute pain to chronic pain, and the modulatory role of dopamine in the NAc changes from being antinociceptive to pronociceptive (Dias et al., 2015). In addition, dysfunction of the dopamine reward response in the NAcSh is known to develop during the late phase of neuropathic pain (Kato et al., 2016). Reduction of locomotor activity accompanied with immobilization of hind limb might also induce the dysfunction of dopamine neurons, as the exercise increases dopamine in the striatum (Hattori et al., 1993) and attenuates the mechanical hypersensitivity induced by immobilization of hind limb (Ishikawa et al., 2021). Taken together, we hypothesized that although this remains to be established, the increased dopaminergic tone and subsequent D2-like receptor activation in the NAcSh attenuates pain in the acute phase but facilitates transition to the chronic phase. When chronic pain develops, the upregulated postsynaptic D2-like receptors with concomitant suppression of dopamine release result in an altered functional state of dopamine neurotransmission to enhance chronic pain. The hypothesized function of postsynaptic D2-like receptors might be applied to mechanical hypersensitivity induced by cast immobilization.

The infusion of the D2-like receptor agonist in the NAcSh of control rats induced mechanical hypersensitivity. Considering that D2-like receptor activation may attenuate acute pain (Taylor et al., 2003; Dias et al., 2015), the reason why acute activation of D2-like receptor signaling in the NAcSh mimicked the chronic phase of mechanical hypersensitivity is not clearly understood. D2-like receptor activation at optimal levels may attenuate acute pain, whereas D2-like receptor activation at high levels may enhance acute pain as well as sensitivity to mechanical stimulation, or vice versa (Pelissier et al., 2006). Future studies to determine the dose-dependent effect of D2-like receptor agonists on acute and chronic pain are required to clarify the relationship between the strength of D2-like receptor activation and pain sensitivity.

Patients with chronic pain are frequently suffered from mood disorders including depression (Yalcin et al., 2014), and the VTA-NAc pathway is involved in pathophysiology of depression (Nestler and Carlezon, 2006). In the model of mechanical hypersensitivity, upregulation of postsynaptic D2-like receptors was observed. Overexpression of D2 receptors in striatal MSNs of adult mice induces the motivational and timing deficits (Drew et al., 2007). In addition, D2-type MSNs are required for aversive learning (Hikida et al., 2010) and optogenetic inhibition of D2-type MSNs decreases motivated behaviors (Soares-Cunha et al., 2016). Taken together these findings, it is possible that inhibition of D2-type MSNs by upregulated D2-like receptors in the NAcSh may contribute to motivational symptoms in chronic pain-induced mood disorders.

### 4.2 Down-regulation of dopamine release by D2-like receptors in immobilization-induced mechanical hypersensitivity

It has been proposed that chronic pain results in the hypodopaminergic state of the VTA-NAc dopamine pathway (Taylor et al., 2016; Gee et al., 2020). Downregulation of the VTA-NAc dopamine pathway and the increased excitability of D2-type MSNs have been implicated in allodynia (Ren et al., 2016) and aversion (Hikida et al., 2010; Danjo et al., 2014), and the inhibition of D2-type MSNs by D2-like receptor activation has been shown to reverse allodynia (Ren et al., 2016) and aversion (Danjo et al., 2014). In agreement with previous studies, mechanical stimulation of the cast-immobilized hind limb induced a decrease in dopamine levels in the NAcSh. Recently, the role of lateral parabrachial neurons in the pons, which consist of the ascending nociceptive pathway and receive input from the spinal cord, in the inhibition of VTA dopamine neurons and dopamine release in the NAcSh in response to noxious stimuli has been reported (Yang et al., 2021). It is possible that lateral parabrachial neurons are involved in the inhibition of the VTA-NAc dopamine pathway under conditions of mechanical hypersensitivity.

In the present study, the decreased dopamine release in response to mechanical stimulation of the cast-immobilized hind limb was mediated through the upregulation of presynaptic D2-like receptor function at dopaminergic terminals in the NAcSh. However, dopamine levels that are under the control of presynaptic D2-like receptors did not correlate with the sensitivity to mechanical stimulation of the cast-immobilized hind limb. This suggests that the upregulation of presynaptic D2-like receptors might act as a compensatory mechanism for the augmented dopamine neurotransmission *via* postsynaptic D2-like receptors, as demonstrated in the dorsal horn as an endogenous antinociceptive system (Abdallah et al., 2015).

### 4.3 Possible therapeutic implications of a D2-like receptor antagonist for chronic pain

The finding that systemic administration of a D2-like receptor antagonist, raclopride, ameliorates mechanical hypersensitivity suggests the clinical usefulness of the D2-like receptor antagonist in chronic pain. Classical antipsychotics with a dominant property of the D2-like receptor antagonist have been used as adjuvant analgesics to treat chronic pain (e.g., chronic headache, fibromyalgia, and diabetic neuropathy); however, their clinical usefulness has been a long-standing controversy (Seidel et al., 2010). From another point of view, antipsychotics with properties of a D2-like receptor antagonist may increase the pain threshold in patients with schizophrenia. Indeed, multiple studies have reported that patients with schizophrenia have an increased pain threshold compared with healthy controls (Bonnot et al., 2009; Stubbs et al., 2015). However, the increased pain threshold seems to be an endophenotype of schizophrenia, which is related to decreased identification and complaints of pain, because antipsychotic-free patients show an increased pain threshold (Bonnot et al., 2009; Stubbs et al., 2015). Taken together, this study clearly demonstrated that blockade of D2-like receptors in the NAcSh is effective in ameliorating mechanical hypersensitivity; however, its beneficial effects may be limited under certain pathophysiological conditions of chronic pain (Seidel et al., 2010). Further studies are required to clarify the therapeutic indications of the D2-like receptor antagonist for specific conditions of chronic pain.

Limitation of the present study is that, first, the influence of gender difference widely known in pain perception (Wiesenfeld-Hallin, 2005) is not evaluated. Large number of studies showed that females are more sensitive than males to pain perception (Mogil, 2012). In female rodents, chronic stress induces mechanical hyperalgesia in proestrus and estrus, but formalin-evoked acute hyperalgesia in metestrus and diestrus (Yang et al., 2020). Contradictory effects of estrous cycle on stress-related nociception may be dependent on the nature of pain and stressor type (Devall et al., 2011; Rahn et al., 2014; Moloney et al., 2016; Yang et al., 2020). In fact, estrogen is known to induce bidirectional effects on pain perception by interacting with different effector systems (Nag and Mokha, 2016; Robinson et al., 2016). Estrogen is also known to affect the density or the ratio of high/low affinity states of D2-like receptors in the striatum (Bazzett and Becker, 1994; Petersen et al., 2021). Estrogen administered in ovariectomized female rats decreased the density of D2-type receptors (Bazzett and Becker, 1994), but controversial results were reported (Hruska and Silbergeld, 1980; Di Paolo et al., 1988; Lammers et al., 1999). A recent PET study in women demonstrated that alteration of circulating estrogen levels during estrous cycle does not affect the availability of D2-type receptors in the striatum (Petersen et al., 2021). Cast immobilization-induced mechanical hypersensitivity might be different between male and female and during estrous cycle, and the differences in this model of mechanical hypersensitivity need to be investigated in future studies. Second, we concluded that the hypersensitivity was due to the upregulation of postsynaptic, not presynaptic, D2-like receptors, based on the consideration of dopamine neurotransmission. Direct evaluation of the expression patterns and the function of postsynaptic D2-like receptors in D2-type MSNs would be required in future studies. Furthermore, it is important to investigate how functional alteration of D2-type MSNs in the NAc contributes to the development of hypersensitivity in neuronal circuits related to chronic pain.

In conclusion, the upregulation of postsynaptic D2-like receptor function in the NAcSh plays a critical role in the mechanical hypersensitivity induced by cast immobilization. Blockade of D2-like receptors is a potential therapeutic strategy for chronic pain accompanying upregulation of postsynaptic D2-like receptors.

## Data Availability

The original contributions presented in the study are included in the article/Supplementary Material, further inquiries can be directed to the corresponding authors.
